# The Measurement and Interpretation of Fibroblast Growth Factor 23 (FGF23) Concentrations

**DOI:** 10.1007/s00223-022-00987-9

**Published:** 2022-06-04

**Authors:** Annemieke C. Heijboer, Etienne Cavalier

**Affiliations:** 1 Endocrine Laboratory, Department of Clinical Chemistry, Amsterdam Gastroenterology Endocrinology & Metabolism, Amsterdam UMC, Vrije Universiteit Amsterdam and University of Amsterdam, de Boelelaan 1117 and Meibergdreef 9, Amsterdam, The Netherlands; 2 Department of Clinical Chemistry, CHU de Liège, University of Liège, 4000 Liège, Belgium

**Keywords:** Phosphate homeostasis, FGF23, Immunoassays, Clinical application, Risk Prediction, CKD

## Abstract

Two decades after the discovery of the hormone FGF23, we know more about phosphate homeostasis as it turned out that FGF23 is the central hormone that regulates this. Hereditary hypophosphatemic rickets and tumor-induced osteomalacia could by then be explained, by autonomous FGF23 production, and the nephrology field was excited by this new marker as it turned out to be independently associated with mortality in people treated by hemodialysis. This led to the development of several immunoassays to be able to measure FGF23 in blood. In the past years we learned that FGF23 is a rather stable peptide, the precision of the assays is acceptable but assays are not standardized and therefore not comparable. This means that reference values and cutoff values need to be assay specific. For several assays reference values have been established and gender and age did not seem of high importance. The phosphate content of the diet, which can be culturally dependent, however, should be taken into account when interpreting results, but to what extent is not totally clear. Currently, clinical application of the immunoassays is established in the diagnosis of hereditary hypophosphatemic rickets and diagnosis and follow-up of tumor-induced osteomalacia. Definite conclusions on the usefulness of the FGF23 measurement in people with CKD either as a marker for risk prediction or a as target for treatment remains to be determined. The latter applications would require dedicated prospective clinical trials, which may take years, before providing answers. To improve the standardization of the FGF23 assays and to shed light on the biological functions that fragments might have we might aim for an LC–MS/MS-based method to quantify both intact and fragmented FGF23. In this literature review we will summarize the current knowledge on the physiological role of FGF23, its quantification, and the clinical usefulness of its determination.

## Introduction

More than 20 years ago Fibroblast Growth Factor 23 (FGF23) was identified for the first time [1]. Although FGF23 was first localized in the brain and expected to play a role in the function of the ventro lateral thalamic nucleus, it soon became clear that FGF23 was a phosphaturic hormone and the causative factor of tumor-induced osteomalacia (TIO) [2]. Since then, numerous reports have highlighted the central role of FGF23 in various diseases and some others have investigated the preanalytical and analytical characteristics of FGF23 determination. In this review we will summarize the current knowledge on the physiological role of FGF23, its quantification, and the clinical usefulness of its determination.

## Metabolism and Role of FGF23

The 32-kDa peptide FGF23 is mainly synthesized in bone cells, specifically in the osteoblasts and osteocytes [3, 4]. FGF23 is transcribed and translated as an inactive, 251-amino acid (AA) peptide, which, after cleavage of the signal peptide (the first 24 AA) gives the intact FGF23 (iFGF23). This peptide can further be cleaved by subtilisin-like pro-protein convertases, such as furin, at a consensus sequence Arg^176^-X-X-Arg^179^ into inactive C- and N- terminal fragments [5–7]. The enzyme GalNAc-T3, coded by the GALNT3 gene, specifically glycosylates FGF23 at Thr^178^. This site-specific O-glycosylation inhibits the action of furin pro-protein convertase and enables secretion of intact active FGF23 [8]. On the other hand, phosphorylation of the kinase FAM20C seems to inhibit glycosylation by GalNAcT3, and thereby promotes cleavage of iFGF23 [9, 10]. In humans, the half-life of iFGF23 is ~ 45–60 min [11].

FGF23, PTH and 1.25 dihydroxyvitamin D (1.25diOHD), the active form of vitamin D, interact one with the other and play an important role in the regulation of phosphate homeostasis [12]. In the kidney, FGF23 binds to the FGF1 receptor and, in the presence of the critical co-receptor α-klotho, suppress the expression of the renal phosphate transporters NaPi2a and NaPi2c, thereby inducing decreased tubular phosphate reabsorption. FGF23 also severely downregulates the vitamin D metabolism by inhibiting CYP27B1, which transforms the inactive 25OHD into 1.25diOHD. Of note, 1.25diOH2D also downregulates its own activation by acting on CYP27B1. FGF23 stimulates CYP24A1 which degrades 25OHD and 1.25diOH into 24,25diOHD and 1.24.25triOHD, which are further transformed into calcitroic acid, an inactive metabolite. PTH also plays an important role in the regulation of phosphate by decreasing the abundance of NaPia2 in the brush border membrane of the renal proximal cells, thus leading to increased phosphaturia [13]. On the other hand, PTH also increase serum phosphate concentration by directly stimulating bone turnover and phosphate release or by indirectly stimulating intestinal phosphate absorption through its stimulatory effect on CYP27B1 activity and 1.25diOH production [14]. The FGFR1-Klotho receptor complex is also expressed in the parathyroid glands and binding of FGF23 leads to a decrease in PTH gene expression and parathyroid cell proliferation [15]. Parathyroid glands also express the VDR and the stimulation of the receptor by 1.25diOHD leads to a decrease in PTH synthesis and secretion and inhibits PTH cell proliferation [16]. Both 1.25diOHD and PTH stimulate FGF23 production in the osteocytes by acting on its promoter [17] and activating the orphan nuclear receptor Nurr1 [18], respectively.

In pathophysiological situations with a primary surplus of FGF23, such as the genetic diseases autosomal dominant hypophosphatemic rickets (ADHR), autosomal recessive hypophosphatemic rickets (ARHR) and X-linked hypophosphatemia (XLH), and tumor-induced osteomalacia (TIO), hypophosphatemia occurs, which may lead to rickets or osteomalacia [19]. On the other side of the spectrum is the genetic disease, hyperphosphatemic familial tumoral calcinosis (HFTC). This disease can be caused by various mutations, in the genes encoding for FGF23, GALNT3, or Klotho. As described earlier, the GALNT3 enzyme is responsible for FGF23 glycosylation which prevents inactivation. Therefore, both GALNT3 and FGF23 mutations lead to decreases in intact FGF23 concentrations. Mutations in the Klotho gene lead to FGF23 resistance. All these mutations result in hyperphosphatemia and ultimately ectopic calcifications [20].

On the other hand, in people suffering from chronic kidney disease (CKD), FGF23 concentrations are increased. This can be explained by multiple reasons. First, since less phosphate is filtered by the glomerulus, serum phosphate concentrations tend to increase, inducing an adaptive increase of FGF23 and an increase of phosphaturia. In the first phase of the disease, this is sufficient to keep serum phosphate concentrations within normal ranges, but as the disease progresses, phosphate concentrations will increase further as does FGF23 [21]. Second, higher FGF23 concentrations observed in subjects with CKD can also reflect tubular dysfunction, independently from GFR [22]. And third, as CKD suppresses klotho expression, FGF23 resistance can be expected [23, 24].

High FGF23 concentrations are thought to induce left ventricular hypertrophy and other cardiovascular pathology although causality has not been proven yet [25, 26].

## Analytical Considerations

### Commercially Available Assays

Currently, circulating FGF23 can be measured using immunoassays. Both intact FGF23 (iFGF23) assays and so called C-terminal FGF23 (cFGF23) assays are available on the market (see Table 1). The intact assays have in common that they claim to exclusively measure the intact protein by using (at least) two antibodies of which one is directed to the N-terminal domain and the other one binds to the C-terminal domain. The cFGF23 assays, also sandwich immunoassays, make use of two antibodies binding to two different epitopes on the C-terminal domain. To date, there are twelve commercially available immunoassays on the market. Ten of these assays measure iFGF23: manual immunoassays from Quidel-Immutopics (currently the 2nd generation) (San Clemente, CA, USA), from Kainos (Tokyo, Japan), from Merck-Millipore (Burlington, MA, USA), from Biomedica (Vienna, Austria), from Minaris Medical (MedFrontier, Tokyo, Japan), from USCN (Wuhan, China), from Avira Bioscience (Santa Clara, CA, USA) and from LSBio (Seattle, WA, USA) and automated chemiluminescence immunoanalyzers from Diasorin (Liaison XL, Saluggia, Italy) and Minaris Medical (CL-JACK system, Japan). Three other commercially available assays are cFGF23 assays which measure the sum of the intact molecule and C-terminal fragments: ELISAs from Quidel-Immutopics, Biomedica, and Avira Biosience. Almost all immunoassays are “research only” kits. Only the automated immunoassay from Diasorin and both FGF23 assays from Biomedica are CE-marked and thereby allowed to use for patient care on the European market. The CL-JACK system, which is hardly available outside Japan, is allowed for in vitro diagnostics on the Japanese market.

**Table 1:** Characteristics of commercially available assays to measure FGF23 according to the package insert of the manufacturers and independently published assay variation

Name of the assay	Manufacturer	IVD/RUO	Automated	Detection	Antibodies	Detection limit	Highest measurable value	Sample volume	Sample recommended	Intra-assay CV	Inter-assay CV	Intra assay CV (literature)	Inter assay CV (literature)
Intact FGF 23 assays													
Human FGF-23 intact	Quidel-Immutopics, San Clemente, California	RUO	NO	2 steps ELISA (HRP)	1 murine monoclonal antibody and 1 affinity purified goat polyclonal antibody	1.5 pg/mL	660 pg/mL	50 µL	Plasma, cell culture	< 4.1%	< 9.1%	< 6% [35], < 10% [27]	First generation: 22–61% [35]; 14–40%[69] Second generation: 5.2% [43], 25.5% at 31.6 ng/mL and 6.2% at 155.6 ng/mL[31], 6.9–19.2% [30]
FGF-23	Kainos, Tokyo, Japan	RUO	NO	2 steps ELISA (HRP)	2 murine monoclonal antibodies	3 pg/mL	800 pg/mL	50 µL	Serum is recommended. heparin and EDTA plasma may be used	< 3%	< 3.8%	< 10% [35], < 10%[27]	< 14% with manual washing step [35]
Human FGF-23	Merck Millipore, Burlington, MA, USA	RUO	NO	2 steps ELISA (HRP)	2 goat polyclonal antibodies	9.9 pg/mL	2400 pg/mL	50 µL	None recommended. Serum, plasma, adipocyte extract, cell culture	< 11%	< 11%	15% at 18 pg/mL [27]	
Intact FGF23	Biomedica Medizinprodukte, Vienna, Austria	CE IVD	NO	1 step ELISA (HRP)	1 monoclonal recombinant anti-human GGF23 antibody and 1 monoclonal mouse anti-human FGF23 antibody	5.4 pg/mL	1600 pg/mL	50 µL	None recommended Plasma (EDTA, citrate, heparın), serum, urine, cell culture supernatant	< 6%	< 8%		
MedFrontier intact FGF23	Minaris Medical, Tokyo, Japan	RUO	NO	CLEIA	2 mouse monoclonal antibodies	10 pg/mL	3000 pg/mL	20 µL	Serum	< 5%	< 5%		
FGF23	USCN (Wuhan, China)	RUO	NO	ELISA (HRP)	“Antibodies specific to FGF23”	15.6 pg/mL	1000 pg/mL	100 µL	Serum, plasma, tissue homogenates, cell lysates, cell culture supernates and other biological fluids	< 10%	< 12%		
Human FGF-23	Avira Bioscience, Santa Clara, CA, USA	RUO	NO	2 steps ELISA (HRP)	“recombinant human FGF-23 and antibodies raised against this protein”	15 pg/mL	2000 pg/mL	100 µL	More recommended. Serum, EDTA plasma	< 8%	< 12%		
Human FGF23	LSBio, Seattle, WA, USA	RUO	NO	2 steps ELISA (HRP)	No information provided	15.6 pg/mL	1000 pg/mL	100 µL	None recommended. Serum, plasma	< 4%	< 6.4%		
LIAISON® FGF 23	DiaSorin, Saluggia, Italy	CE IVD	YES	CLIA	3 monoclonal antibodies, 1 directed against the N-terminal portion of iFGF23, another, labeled with fluoresceine, directed against the C-terminal portion, and the 3rd one, bound with isoluminol, directed against fluoresceine	5 pg/mL	5000 pg/mL	150 µL	EDTA plasma	< 4%	< 8%		< 2.5% at 50 and 242 pg/mL) [31], 5.8–7.1% [30]
CL-JACK FGF23	Minaris Medical, Tokyo, Japan	IVD Japan*	YES*	CLEIA*	2 mouse monoclonal antibodies*	5 pg/mL*	10,000 pg/mL*	10 µL*	Serum*	< 10%*			< 3.5%[33]
C-terminal FGF 23 assays													
MicroVue Human FGF-23 (C-term)	Quidel- Immutopics, San Clemente, California	RUO	NO	2 steps ELISA (HRP)	Two affinity purified goat polyclonal antibodies	1.5 RU/mL	1400 RU/mL	100 µL	Plasma or cell culture media	< 2.4%	< 4.7%	< 5%[35], < 10% [27]	First generation: < 16%[35], ~ 10% [69] Second generation: 7.2% [43]
FGF23 (C-terminal)	Biomedica Medizinprodukte, Vienna, Austria	CE IVD	NO	1 step ELISA (HRP)	1 polyclonal rabbit anti-human FGF23 and 1anti-human FGF23 antibody	0.08 pmol/ (0.6 pg/mL)	20 pmol/L (150.4 pg/mL)	50 µL	Serum, EDTA plasma, citrate plasma, heparin plasma	< 10%	< 12%		
Human FGF 23 C-terminal peptide	Avira Bioscience, Santa Clara, California	RUO	NO	2 steps ELISA (HRP)		15 pg/mL	16,000 pg/mL	100 µL	More recommended. Serum, EDTA plasma	< 8%	< 12%		

*CLIA* chemiluminescence immunoassay, *CE IVD* European Mark for In Vitro Diagnostic use in Diagnostic, *RUO* For research use only, not to be used for diagnostic purposes, *ELISA* Enzyme-Linked Immunosorbent Assay, *HRP* Horseradish peroxidase, *CLEIA* Chemiluminescence enzyme immunoassay, *NP* not provided, *CV* coefficient of variation

*Information only available in Japanese and freely translated

Some characteristics about these assays, from the package insert of the manufacturer, are shown in Table 1. Although quite some independent reports have been published on the analytical and pre-analytical aspects of FGF23 assays, this is certainly not the case for all available assays. Especially surprising is that analytical and pre-analytical data about the CE-marked assays from Biomedica are scarce. In the coming paragraphs the available analytical and pre analytical data from the literature are discussed and the biological variation, reference, and cutoff values are summarized.

### Precision

Precision stated in the package insert of the manufacturer is not always repeatable in the clinical laboratory using clinical samples. This is the reason that laboratories should verify assays before using them. Not for all available FGF23 assays precision data determined in clinical laboratories are available in literature, however, especially for the assays that are on the market for a longer period of time, data are available and shown in Table 1. According to the biological variation of FGF23 (see below), the desirable precision for assays used in a clinical context should be lower than 7%.

### Comparability of Assays

Methods comparisons between the c-term FGF23 assays are currently not available in literature. Comparisons between the different iFGF23 assays are available but unfortunately results do not correspond with each other (see Table 2). As no international standard for iFGF23 is available, it is currently not possible to standardize the assays and harmonization of the different assays is not initiated up till now. Method comparisons show we have to deal with substantial standardization differences between the assays. The method comparisons performed with some sort of regression analysis and expressed in formula are shown in Table 2. The Immutopics iFGF23 assay gives in absolute concentrations lower results than the Kainos iFGF23 assay in healthy subjects, people treated by hemodialysis, people with TIO and in children [27–29]. The Immutopics iFGF23 assay measured ~ 20% lower than the Liaison assay in two studies [30, 31]. The CL-Jack iFGF23 assay was shown to measure ~ 25% lower than the Kainos iFGF23 assay, in samples from people with hypophosphatemia which was a confirmation of an earlier method comparison of the CL-Jack method compared to the Kainos iFGF23 assay, performed in samples with high concentrations [32, 33]. To make it more complicated, the standardization differences are not equal for the whole concentration range and/or between patient groups. For instance, the Immutopics iFGF23 assay gave 55% lower results than the Kainos iFGF23 assay in healthy subjects but 90% lower results in people treated by hemodialysis [27]. The same issue was shown in a method comparison between the Millipore assay and the Kainos iFGF23 assay; the Millipore assay measured iFGF23 15% lower than the Kainos assay in healthy subjects yet 50% lower in people treated by hemodialysis [27]. In addition, these comparison studies often show very low correlation coefficients between different iFGF23 assays [28, 29, 33]. This might be due to variation in the epitopes of the antibodies used in the different iFGF23 assays and in the different cFGF23 immunoassays. This means that we have to conclude that not only standardization differences hamper the comparability between iFGF23 assays, but that individual differences or differences between patient groups exist as well. This suggests that harmonization or standardization will be a challenging task.

**Table 2:** Comparison of different methods for iFGF23 quantification

Reference	Regression analysis	$y$	$x$	*n* =	Sample type	Population	Equation
Kato et al. [32]	Linear regression	CL-JACK	Kainos	380	Serum	Healthy subjects and subjects suffering from phosphate metabolism diseases	$y=0.76\times x-0.32$
van Helden et al. [30]	Passing & Bablok	Immutopics	Liaison XL	140	EDTA plasma	Healthy subjects and subjects suffering from phosphate metabolism diseases	$y=0.78\times x-10.99$
Smith et al. [27]	Passing & Bablok	Immutopics	Kainos	31	EDTA plasma	Healthy subjects	$y=0.45\times x+0.45$
Smith et al. [27]	Passing & Bablok	Immutopics	Kainos	36	EDTA plasma	Subjects treated by dialysis	$y=0.10\times x+108.6$
Smith et al. [27]	Passing & Bablok	Millipore	Kainos	31	EDTA plasma	Healthy subjects	$y=0.85\times x-5.39$
Smith et al. [27]	Passing & Bablok	Millipore	Kainos	36	EDTA plasma	Subjects treated by dialysis	$y=0.52\times x+56$
Smith et al. [27]	Passing & Bablok	Immutopics	Millipore	31	EDTA plasma	Healthy subjects	$y=0.57\times x+2.06$
Smith et al. [27]	Passing & Bablok	Immutopics	Millipore	36	EDTA plasma	Subjects treated by dialysis	$y=0.21\times x+107.6$
Souberbielle et al. [31]	‘Regression’	Immutopics	Liaison XL	87	EDTA plasma	Subjects from daily routine practice	$y=0.78\times x+0.6$
Shimizu et al. [33]	Not mentioned	CL JACK	Kainos	182	Serum	Subjects with chronic hypophosphatemia	$y=0.82\times x+9.42$
Cavalier E (unpublished results, 2021)	Passing & Bablok	Liaison XL	Kainos	69	EDTA plasma	Subjects from daily routine practice	$y=0.82\times x-1.53$

### Interferences

The FGF23 assays can suffer from interferences like heterophilic antibodies as it is the case with all immunoassays. It is of special interest that the FGF23 assays can specifically be influenced by the new treatment for X-linked hypophosphatemia, burosumab. Burosumab is a monoclonal antibody that binds intact FGF23 at the N-terminal domain. Although being an effective treatment, this antibody interferes in several FGF23 assays, leading to very high concentrations in some cFGF23 assays and falsely low or high concentrations in some iFGF23 assays [34].

## Preanalytical Considerations

### Matrix

While the Immutopics cFGF23 and iFGF23 can be measured in EDTA plasma only, as measurement in serum using these assays leads to low or undetectable results, the Kainos iFGF23 assay can be measured in both EDTA plasma and serum [27, 35, 36]. The Liaison iFGF23 assay measures 50% lower in serum than in the recommended EDTA plasma [31]. Manufacturers recommend in their manuals different types of tubes, and in some assays all matrices are said to be appropriate to use. This is partly summarized by Fauconnier et al. [37]. Besides the inherent relative differences between the matrices, FGF23 is more stable in EDTA plasma than in serum [38, 39]. We would therefore recommend to use EDTA plasma to make result as comparable as possible.

### Stability

Stability can be divided into short-term stability (pre-centrifugation stability (centrifugation < 1 h), delayed centrifugation (centrifugation after 8 h), and delayed storage stability (after centrifugation), long-term stability in the freezer, and the stability after freeze–thaw cycles.

The short-term stability has been extensively described in literature and summarized in 2019 by Dirks et al. [40]. After Smith et al. showed that the iFGF23 assay suffered from immediate pre-centrifugation proteolysis and that protease inhibitors were necessary [41], later studies demonstrated that this was only the case for the first generation iFGF23 assays [40]. Currently, coating the EDTA collection tubes with protease inhibitors is not necessary for the current Immutopics, Kainos, Millipore, and Diasorin iFGF23 assays. However, centrifugation should not be delayed as FGF23 concentrations decline after delayed centrifugation for 6–8 h [40]. After centrifugation, iFGF23 in plasma or serum is stable [40].

Long-term stability is studied less extensively. One study demonstrated the stability of iFGF23 (Kainos assay) and cFGF23 (2^nd^ generation Immutopics assay) in 10 plasma samples stored at – 80 °C for 40–60 months and showed a small decline in concentrations [36]. Another study concluded that no significant decline was seen in iFGF23 (Kainos assay) concentrations in 6 serum samples after 6 years of storage at – 80 °C, albeit that the 20 samples in the lower concentration range showed a decline of ~ 48% [42].

Freeze–thaw cycles have been studied using different assays. Up to five freeze–thaw cycles had no relevant effect on iFGF23 and cFGF23 concentrations in serum and EDTA plasma (measured using 2nd gen Immutopics assays, the CL-Jack assay, and the Kainos assay) [33, 36, 42, 43].

### Diurnal Variation

FGF23 seems to have a diurnal variation in both women and men [44–46]. One study concluded that both iFGF23 (Kainos assay) and cFGF23 (Immutopics assay) show diurnal variation [45] yet another study concluded that only iFGF23 (Immutopics assay) but not cFGF23 (Immutopics assay) displays a diurnal variation [44]. Of note, both studies had a relatively low number of blood withdrawal time points during the day (three and four, respectively). The study of Swanson et al. [46] was designed to determine a 24 h profile by measuring iFGF23 every 2 h during 24 h. iFGF-23 was shown to be rhythmic (*p* < 0.001), with the estimated acrophase at 08:30 h (relative clock time) and the nadir occurred at 20:30 h, although there was quite some inter-individual variation [46]. Unfortunately, cFGF23 was not measured in this study.

### Impact of Diet on FGF23

A high phosphate diet leads to higher iFGF23 and cFGF23 concentrations compared to a phosphate restricted diet in healthy subjects and a low phosphate diet seems to decrease iFGF23 (but not cFGF23) concentrations in people with chronic kidney disease (CKD) [45, 47]. As phosphate absorption is higher from meat-based than from plant-based protein sources, one could imagine that vegetarians would have a lower FGF23 concentration. If so, this would have implications for reference values and cut-of values. Indeed, in people with advanced CKD iFGF23 was lower after a vegetarian diet for 7 days. An Indian study in the general population however did not show differences in iFGF23 between urban non-vegetarians, urban vegetarians and rural vegetarians irrespective of age, sex, presence of diabetes, and BMI. [48]. On the other hand, a study in African adults living in Nigeria and African Americans living in the USA showed a higher urinary phosphate excretion and higher cFGF23 levels in the African Americans living in the USA, presumably due to differences in dietary habits [49].

## Biological Variation, Reference Values, Cutoff Values

### Biological Variation

Biological variation is the sum of the within-subject variation and the between-subject variation and important to calculate the reference change value (formerly called least significant change). A reference change value can be used in the follow-up of patients to determine whether there is a significant increase or decrease of the biomarker, in this case of FGF23. Up to now three studies investigated the biological variation of FGF23 in healthy subjects. Two studies used the Liaison XL for iFGF23 measurement and both studies found a within-subject variation of 14% and a reference change value of ~ 40% [50, 51]. Another study measured these parameters using the Immutopics assays and found a within-subject variation of 18.3% and a reference change value of 54% for iFGF23 and a within-subject variation of 8.3% and a reference change value of 25% for cFGF23 [44]. Biological variation using the iFGF23 assay of Immutopics was determined in stable hemodialyzed patients as well, showing a within-subject variation of 17.2% and a reference change value of 48% [52]. Together, these studies suggest that, regarding biological variation and the reference change value, there does not seem to be a relevant difference between iFGF23 assays and between healthy and dialyzed populations.

### Reference Interval

In the past 20 years several groups conducted studies to determine references intervals for FGF23. Some laboratories might have established reference intervals that are only being used for internal purposes, while other published these results. We summarized the in literature available reference intervals including the sex, age, and other information about the subject and immunoassay that was used (Table 3). As the available assays are not standardized, it is crucial to use assay-dependent references values. In three studies men had statistically significant higher FGF23 concentrations than women although absolute differences were small [30–32]. In one study the opposite was seen, and in other studies no differences were observed or gender was not mentioned [44, 48, 53]. Also in children, FGF23 concentrations do not seem to be gender-dependent [54–56]. It seems therefore unlikely that gender specific reference intervals are needed. Age dependent reference intervals for adults are probably not necessary as well. Several studies found age dependent differences in FGF23 concentrations, yet these disappeared after exclusion of subjects with a lower eGFR [31, 32, 44, 53]. In children cFGF23 concentrations are somewhat higher than in adults. During childhood FGF23 is somewhat higher in the youngest children (< 2 year) and in those between 12 and 15 years of age [54, 55].

**Table 3:** An overview of established references intervals for FGF23

Study	Participants	Male/female	Age (years)	Assay	Reference values	
Kato et al. [32]	380 healthy Japanese participants	198/182	20–79	iFGF23 (CL-Jack system)	16.1–49.3 pg/mL Men significantly higher than women (men mean [IQR] 32.5 [29.2–38.2]; female: 30.2 [25.1–36.1]). No differences observed between age groups (decades)	
Souberbielle et al. [31]	908 healthy French subjects	482/426	18–89 (39.7 ± 18.6)	iFGF23 (Diasorin)	22.7–93.1 ng/L. Men significantly higher than women (60.3 ± 17.6 ng/L versus 55.2 ± 17.2 ng/L). Subjects > 60 years slightly higher FGF23 (63 ± 19.3 ng/L) than 30–59 years (56.1 ± 17.3 ng/L) and than 18–29 years (56.9 ± 17.3 ng/L). 60 + also had a lower eGFR. After exclusion of the subjects with an eGFR of < 75 mL/mn there was no effect of age anymore	
Van Helden et al. [30]	153 putative healthy subjects	81/72	Males: 18–81 Females: 21–78	iFGF23 (Diasorin)	Males: 23.7–92.5 pg/mL females: 21.2–89.4 pg/mL	
Yamazaki et al. [53]	104 healthy controls	30/74	Males: 24–59 Females: 21–63	iFGF23 (Kainos)	8.2–54.3 pg/mL (range) Age- or gender-dependent differences were not observed	
Anand et al. [48]	1192 study participants from India	45%/55%	47 (± 14)	iFGF23 (Immutopics)	41 ± 18 pg/mL Median FGF23 in woman somewhat higher than in men (39.7 versus 37.1 pg/mL)	
Smith et al. [44]	170 healthy individuals	60%/40%	56 (± 13)	iFGF23 and 2nd gen cFGF23 (both Immutopics)	iFGF23: 11.7–48.6 pg/mL cFGF23: 21.6–91 RU/mL No association with gender or age	
Jialal et al. [56]	100 volunteers with eGFR > 60 ml/min/m2	Not mentioned	Not mentioned	cFGF23 (Immutopics)	21–424 RU/mL	
Imel et al. [29]	118 normophosphatemic individuals (of which 8 children) 60 children	Not mentioned	20–83 (the children 2–14) Children: 1–17	iFGF23 (Kainos) iFGF23 (Immutopics) cFGF23 (Immutopics)	iFGF23 (K): < 71 pg/mL iFGF23 (I): < 51 pg/mL cFGF23 (I) adults: < 149 RU/mL cFGF23 (I) children: < 142 RU/mL	
Fischer et al. [54]	424 healthy Caucasian children, adolescents and young adults	221/203	0.1–21 years	2nd gen cFGF23 (Immutopics)	Age	Reference interval (RU/mL)
					≤ 1	43–324
					2	39–235
					3	36–192
					4	34–163
					5	33–145
					6	33–135
					7	34–129
					8	35–126
					9	36–126
					10	37–128
					11	37–132
					12	37–136
					13	36–139
					14	35–140
					15	34–136
					16	32–127
					17	29–114
					18	26–100
					≥ 19	22–85

As the amount of phosphate in the diet might influence the FGF23 concentrations, and as the phosphate content differs per country, it might be necessary to determine reference values per country in order to take dietary habits into account when interpreting FGF23 concentrations.

### Cut-off Values to Detect FGF23-Induced Hypophosphatemia

To determine which persons with a low serum phosphate concentration have a FGF23-induced hypophosphatemia an FGF23 cut-off level is useful. When using the upper limit of the reference interval as cut-off level, the Kainos iFGF23 assay showed a 100% sensitivity, the Immutopics cFGF23 assay (1st generation) a 92% sensitivity and the Immutopics iFGF23 assay (1st generation) a 38% sensitivity in detecting all hypophosphatemic patients with a confirmed TIO according to Imel et al. [29]. In 2008, Endo et al. reported on optimal cut-off values for the clinical usefulness of FGF23 measurements in people with hypophosphatemia. They proposed the following cut-off values: a serum phosphate concentration of < 2.5 mg/dL and an iFGF23 concentration (measured using the Kainos assay) of > 30 pg/mL indicates the presence of a disease caused by excess FGF23 such as TIO or XLH [57]. For children and the phosphate cut-off values should be somewhat higher depending on the age < 3.5 or < 4.5 mg/dL. These diagnostic phosphate and iFGF23 cut-off values were adopted in the guidelines of the Japanese Endocrine Society and the Japanese Society for Bone and Mineral Research [58]. Upon the development of an automated iFGF23 immunoassay (CL-Jack), Shimizu et al. determined the cut-off value in people with hypophosphatemia with this new iFGF23 assay, which turned out to be 25 pg/mL, leading to a sensitivity and specificity of 100% [33]. Recently, Ito et al. verified whether the cutoff value of 30 pg/mL could also be applicable for the CL-Jack iFGF23 assay and concluded that it leads to a sensitivity of 100% and specificity of 82% for FGF23-related hypophosphatemic rickets with and without vitamin D deficiency [59]. The four initially classified non-FGF23 related hypophosphatemia patients with an increased FGF23 concentration (leading to the 82% specificity) included 2 patients who in retrospect might have had FGF23-related hypophosphatemia due to ectopic overproduction of FGF23 by a neuroendocrine tumor. When these patients were added to the FGF23-related hypophosphatemia group, the specificity improved to 90% [59]. Up to now, in the literature studying cut-off values only serum phosphate concentrations are taken into account. It might be worthwhile to add the tubular maximum reabsorption of phosphate per glomerular filtration rate (TmP/GFR) to estimate renal phosphate wasting in future studies to improve the selection of patients with FGF23-induced hypophosphatemia. It would be of additional value to determine cut-off levels for other immunoassays than the Kainos and CL Jack assays as well.

## Indications for Measuring FGF23

In the last decades FGF23 has proven its worth in the diagnosis of TIO and hypophosphatemic rickets [60, 61]. For some specific cases of TIO, venous FGF23 sampling can be used for localization of the tumor. FGF23 can also be used in the follow-up after resection of the tumor. Hypophosphatemia due to renal phosphate loss in a setting of normal or low PTH is an accepted indication for FGF23 measurement [61]. In the clinical exploration of a hypophosphatemia in patients presenting high phosphaturia, PTH, and FGF23 are useful to differentiate causes linked to hyperparathyroidism and other diseases linked with PTH increase from FGF23-mediated forms (XLH, ADHR, ARHR1) [62]. The FGF23 concentration can distinguish between proximal renal tubule dysfunction such as Fanconi syndrome (low FGF23 concentration) or FGF23-dependent causes such as TIO (normal or elevated FGF23 concentrations) [63]. Interestingly, solid tumors like neuroendocrine tumors including small cell lung cancer of the lung or prostate and also hematologic malignancies are able to produce FGF23 and could lead to FGF23-related hypophosphatemia [59, 63]. This might be a group of patients where FGF23 could be of additional value to measure when hypophosphatemia occurs.

Although FGF23 is increased in CKD, with extremely high concentrations in people treated by dialysis, and these FGF23 concentrations are independently associated with mortality and related to cardiovascular events [64], to date there is no clear indication to measure FGF23 in people with CKD [22, 26, 37]. More studies are needed to show the usefulness of the FGF23 measurement or serial measurements in these people as a marker for risk prediction and to identify high-risk patients [26, 65]. Of note, Isakova et al. have shown that, if FGF23 concentrations were stable over time in the majority of CKD patients, those presenting a rapid increase in FGF23 were at higher risk of death [65]. As it is still unknown whether directly targeting and lowering FGF23 leads to an improved outcome, we do not know yet whether measuring the concentrations to target therapy is of additional value, and would improve clinical outcome [26, 66, 67].

## Future Perspectives

The development of several FGF23 immunoassays improved the possibility for diagnosing and follow-up of people with hereditary hypophosphatemic rickets and TIO. Definite conclusions on the usefulness of the FGF23 measurement in people with CKD remains to be established and will only be answered when trials with FGF23 targeting interventions will be performed. Nevertheless, FGF23 immunoassays made it possible to study and firmly establish the association of (serial) FGF23 concentrations with clinically relevant endpoints, such as mortality and cardiovascular events. This might lead to FGF23 as a biomarker for risk prediction to identify high-risk patients.

The majority of the currently available immunoassays are ‘research only’ kits, thereby not allowed to use for patient care. Preferably, these assays should be marked for IVD use. From some of the CE-marked assays no analytical data are published, this information gap should be filled. An external quality assessment scheme for FGF23 and harmonization or standardization of the measurements would also help to improve the quality.

The immunoassays that are currently used to measure FGF23 do not measure and quantify both the fragments and the intact hormone separately. In addition, regarding post-translational modification like phosphorylation and glycosylation of the FGF23 protein we are ignorant, both on its impact on assay performance and its clinical relevance. A step forward could therefore be the development of an LC–MS/MS method to quantify FGF23, both intact and fragmented and focusing on possible phosphorylation and glycosylation. Post-translational modifications might affect biological functionality in the physiological situation. A method able to separately detect intact FGF23 and its fragments would shed light on the differences in method comparisons that are observed and also shed light on the biological functions that fragments might have [68]. In addition, such an LC–MS/MS method would be a potential reference method which, together with commutable standard material, could be helpful in the standardization of all available FGF23 methods. FGF23 concentrations are very low, so to develop such a method with the current LC–MS/MS machines is challenging but if possible certainly worthwhile.
